# Magnetic Characteristics of Ni-Filled Luminescent Porous Silicon

**DOI:** 10.3389/fchem.2019.00041

**Published:** 2019-01-29

**Authors:** Petra Granitzer, Klemens Rumpf, Peter Poelt, Michael Reissner

**Affiliations:** ^1^Institute of Physics, University of Graz, Graz, Austria; ^2^Institute for Electron Microscopy, University of Technology Graz, Graz, Austria; ^3^Institute of Solid State Physics, Vienna University of Technology, Vienna, Austria

**Keywords:** porous silicon, photoluminescence, metal deposition, magnetic nanostructures, magnetic behavior

## Abstract

The aim of the presented work is to combine luminescent porous silicon (PSi) with a ferromagnetic metal (Ni) to modify on the one hand the photoluminescence by the presence of metal deposits and on the other hand to influence the optical properties by an external magnetic field. The optical properties are investigated especially with respect to the wavelength-shift of the photoluminescence due to the metal filling. With increasing metal deposits within PSi the photoluminescence peak is blue-shifted and furthermore an increase of the intensity is observed. Photoluminescence spectra of bare PSi show a maximum around 620 nm whereas in the case of Ni filled samples the peak is blue-shifted to around 580 nm for a deposition time of 15 min. Field dependent magnetic measurements performed with an applied field parallel and perpendicular to the surface, respectively, show a magnetic anisotropy which is in agreement with a thin film. This film-like behavior is caused by the interconnected Ni structures due to the branched porous silicon morphology. The coercivity increases with increasing metal deposition from about 150 Oe to about 450 Oe and also the magnetic anisotropy is enhanced with the growth of metal deposits. Within this work the influence of the magnetic metal filling on the optical properties and the magnetic characterization of the nanocomposites are discussed. The presented systems give not only rise to optoelectronics applications but also to magneto optical integrated devices.

## Introduction

Porous silicon in the nanoporous regime with pore diameters of 2–5 nm is known to emit light in the visible since 1990 (Canham, 1990) and it is still under intense investigation (Joo et al., 2016). The light emission can be classified mainly in three bands, the red-, the blue, and the infrared band (Canham, 1995). The origin of the red emission is explained by quantum confinement (Lehmann and Gösele, 1991), the blue emission occurs in oxidized samples (Kanemitsu et al., 1993) and is attributed either to defects in SiO_2_ (Ito et al., 1992) or to OH groups adsorbed on structural defects in SiO_2_ (Tamura et al., 1994). The infrared luminescence which is investigated less extensive is explained by mid-gap dangling bonds on the silicon nanocrystals (Koch et al., 1992).

Electroluminescence of porous silicon has been investigated (Koshida and Koyama, 1992) shortly after the observation of its photoluminescence (Canham, 1990), offering a similar spectrum to the light emission. The high surface area of this nanostructured material which depends on the morphology (nanoporous silicon ~1,000 m^2^/cm^3^, mesoporous silicon ~100 m^2^/cm^3^, macroporous silicon ~1 m^2^/cm^3^) makes it suitable for pore filling using various materials with specific properties which opens the possibility to fabricate new nanocomposite materials with desired physical behavior. Metal filling of porous silicon firstly has been used in the 1990s to improve electrical contact to enhance the efficiency of the electroluminescence (Koshida et al., 1993; Li et al., 1994).

The main intention is to enhance the quantum yield of the photoluminescence. The efficiency of the luminescence generally is in the range of 1–10% without any further treatment of the samples. In the case of electroluminescence it is even reduced due to the absorption in the semitransparent metal layer. To enhance the luminescence of porous silicon various post-treatments of the samples have been performed such as high pressure water vapor annealing (HWA) a technique which is well-controlled (Gelloz et al., 2005) and can be used to get stable and highly efficient visible light emission. In this case the quantum efficiency is higher than 23%. Furthermore, it has been shown that this method leads to sufficiently passivated surfaces with low non-radiative defect density resulting in a stabilization of the samples. A further approach to influence the luminescence is the exploitation of surface plasmons of metal particles on the porous silicon surface as e.g., Au particles (De la Mora et al., 2014). Generally an emitter in the direct vicinity of a metal-dielectric interface couples to the plasmon mode because of the high optical density of states (Huck and Anderson, 2016). A blue shift of the porous silicon photoluminescence has been observed with decreasing particle size of Au nanoparticles which are deposited within the porous silicon (Amran et al., 2013).

Also the incorporation of Ni particles has been used to create new luminescent centers to achieve a luminescence enhancement (Amdouni et al., 2015). In the case of the utilization of magnetic particles not only the plasmon resonance of the metal can be used but also the magnetic properties can be utilized to explore magneto-optical properties of the nanocomposite. Systems for magneto-optical devices should offer on the one hand non-reciprocity which means that the time inverse symmetry is broken and on the other hand they should exhibit a memory effect. In the case of ferromagnetic structures the data can be memorized (Hu et al., 2016). Therefore, it is important to overcome a superparamagnetic behavior. Ni particles have been investigated with respect to the plasmon resonance showing its dependence on the size and shape but also on the surrounding medium (Sharma et al., 2017). This work aims to merge the optical properties of luminescent porous silicon and the magnetic properties of deposited magnetic nanostructures. Therefore, Ni is deposited within the porous silicon layer to exploit the plasmon resonance of the metal deposits to influence, especially to increase the photoluminescence. The photoluminescence as well as the magnetic properties are investigated with respect to the metal filling.

## Experimental Methods

**Figure 1:**
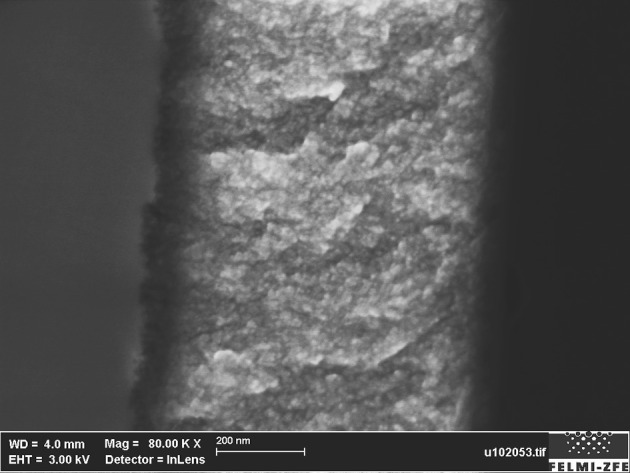
Cross-sectional SEM image showing a microporous layer of about 800 nm.

The porous silicon is fabricated by anodization in a double tank cell of a moderately doped p-silicon wafer (8–12 Ωcm). The electrolyte consists of a hydrofluoric acid solution, consisting of distilled water, HF and ethanol in the ratio 1:1:2. A current density of 10 mAcm^−2^ is applied for 15 min resulting in a microporous layer with a thickness of about 800 nm (Figure 1). A mean pore diameter of about 5 nm is estimated from the optical investigations in using the following equation (Ossicini et al., 2003):

$$\begin{array}{l} E=\;E_{g}+\frac{h^{2}}{8d^{2}}\;\left[\frac{1}{m_{e}^{{\;}^{*}}}+\;\frac{1}{m_{h}^{{\;}^{*}}}\right] \end{array}$$

E, peak position of the photoluminescence

E_g_, energy band gap of bulk silicon

h, Planck's constant

${\text{m}}_{\text{e}}^{*}$, effective mass of electron

${\text{m}}_{\text{h}}^{*}$, effective mass of hole

The metal deposition (Ni) within the porous layer is performed by pulsed electrodeposition in using in the case of Ni a solution consisting of NiCl_2_ and NiSO_4_, whereat H_3_BO_3_ acts as buffer. The Ni-deposition is carried out on different porous silicon samples offering an equal morphology by varying the deposition time between 5 min and 15 min. A current density of 10 mA/cm^2^ with a frequency of 2 Hz is applied. The size of the deposited metal structures corresponds to the pore size of the porous silicon. Due to the interconnected morphology of microporous silicon also the Ni deposits offer interconnections rather than separated particles. From the knowledge of the Energy Dispersive X-Ray (EDX) spectra taken along the cross-section of porous silicon samples filled for 15 min with Ni the metal is deposited down to the pore tips.

First the optical properties of the luminescent PSi are investigated, especially the photoluminescence and the corresponding decay times, second Ni is electrochemically deposited in the porous silicon samples and subsequently the nanocomposite specimens are characterized optically and magnetically. The samples are structurally characterized by scanning electron microscopy (SEM) and Energy Dispersive X-ray diffraction (EDX). The magnetic measurements are performed with a vibrating sample magnetometer (PPMS, Quantum Design).

## Results and Discussion

**Figure 2:**
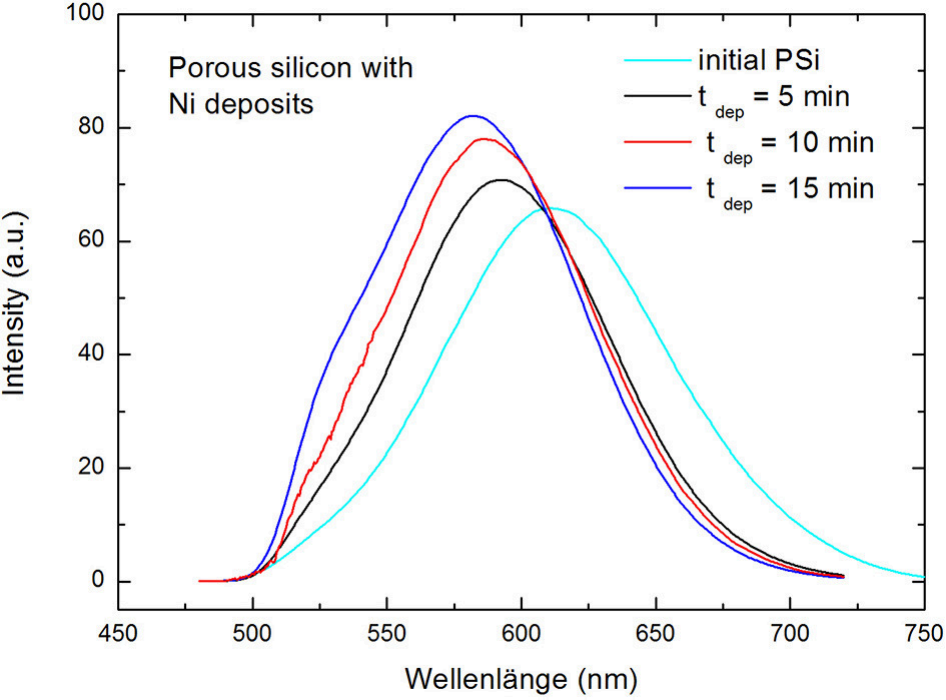
Blue-shift of the photoluminescence peak with increasing metal deposition time in comparison to initial porous silicon. An enhancement of the photoluminescence intensity with increasing metal deposition is also observed.

The optical properties are investigated especially with respect to the wavelength-shift of the photoluminescence (PL) due to the metal filling. Photoluminescence spectra of the used bare PSi show a maximum around 620 nm whereas in the case of Ni filled samples the peak can be blue-shifted to around 580 nm in using a metal deposition time of 15 min and the luminescence intensity is increased. Figure 2 shows the comparison of the photoluminescence of initial porous silicon and Ni-filled samples with different metal deposition times. After a deposition time of 15 min EDX-spectra evidence Ni is deposited down to the bottom of the porous layer. All used samples offer the equal morphology which has been proved by investigating the photoluminescence before the Ni deposition. For the Ni deposition with various times always a current density of 10 mA/cm^2^ with a frequency of 2 Hz is applied.

**Figure 3:**
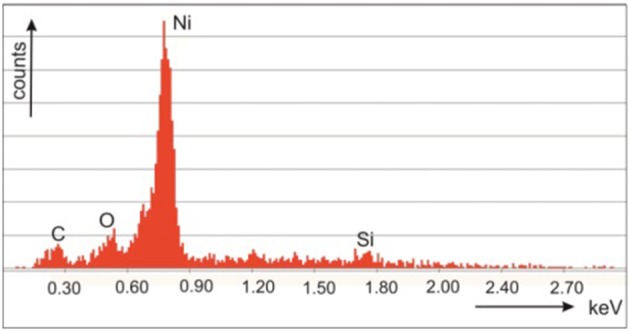
EDX-spectrum at the cross-section of a Ni filled microporous silicon sample.

**Figure 4:**
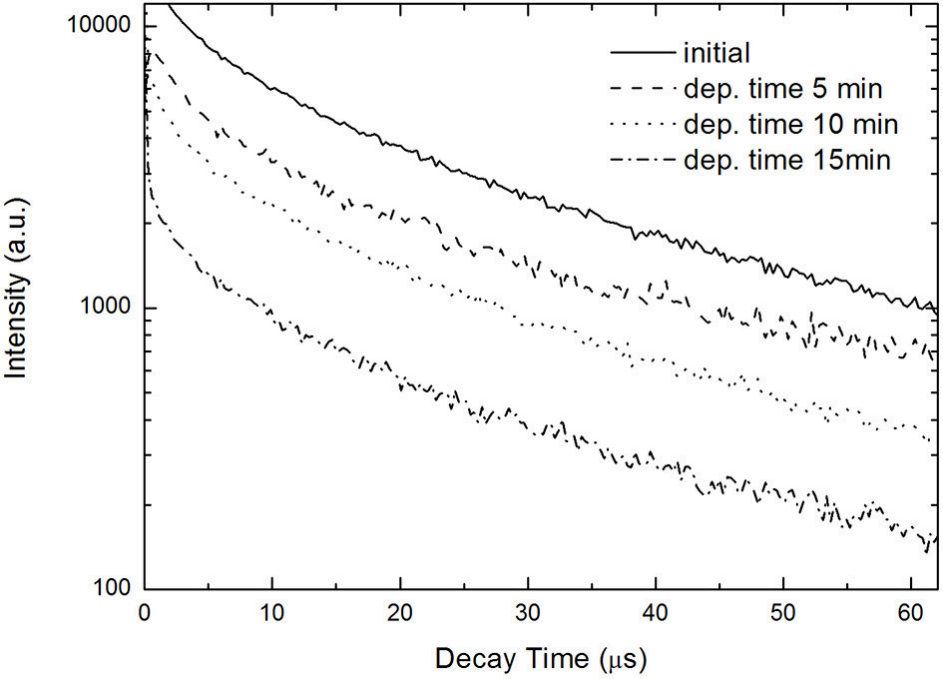
Decay times of initial porous silicon in comparison to Ni-filled porous silicon. The measurements show a decrease of the decay time with increasing metal deposition time from about 300 μs to about 100 μs.

In Figure 3 a cross-sectional EDX spectrum showing the Ni content of a sample prepared with a 15 min deposition time is presented. The decay times of the samples are measured with an excitation wavelength of 440 nm. The measurements show that the decay times of Ni-filled samples are faster than for the bare porous silicon (Figure 4) which indicates a decrease in the radiative life time with increasing metal filling. The decay time for initial porous silicon is around 300 μs, for the sample filled with Ni for 5 min around 250 μs, for a sample filled with Ni for 10 min about 150 μs and for a 15 min Ni filled sample about 100 μs.

**Figure 5:**
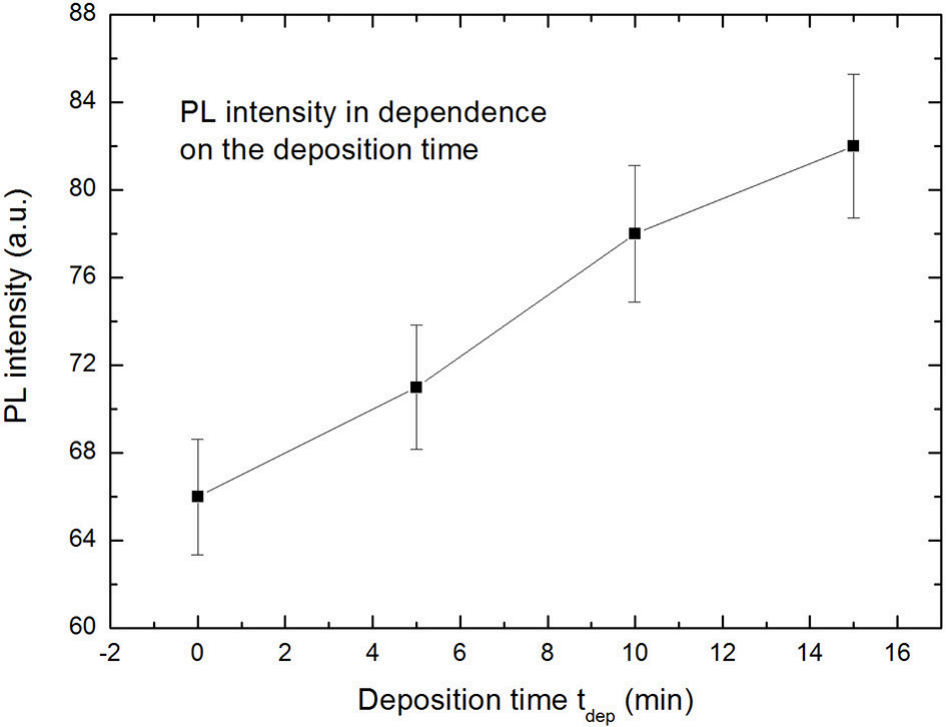
With increasing metal (Ni) deposition time an increase of the photoluminescence intensity is observed which is attributed to the coupling between the emitter and the deposited metal structure (Granitzer et al., 2018).

In addition to the blue-shift of the PL-peak an increase of the luminescence intensity with increasing deposition time is observed which is shown in Figure 5. This result strongly indicates the involvement of Ni plasmons.

Concerning the magnetic properties of the nanocomposite the embedded Ni structures can be superparamagnetic from the size of the pore diameters which are around 5 nm (gained from optical measurements). Small separated magnetic particles of a few nanometers in size offer superparamagnetic behavior which can be figured out by temperature dependent measurements. From such measurements the transition temperature between superparamagnetic and blocked state can be determined. The transition temperature strongly depends on the particle size as well as on the proximity of the particles and thus their magnetic coupling. The investigated samples offer a branched morphology and therefore the achieved deposits tend to be interconnected and thus do not offer necessarily a superparamagnetic behavior. Temperature dependent magnetization measurements of the samples give no hint for superparamagnetism of the nanocomposite.

**Figure 6:**
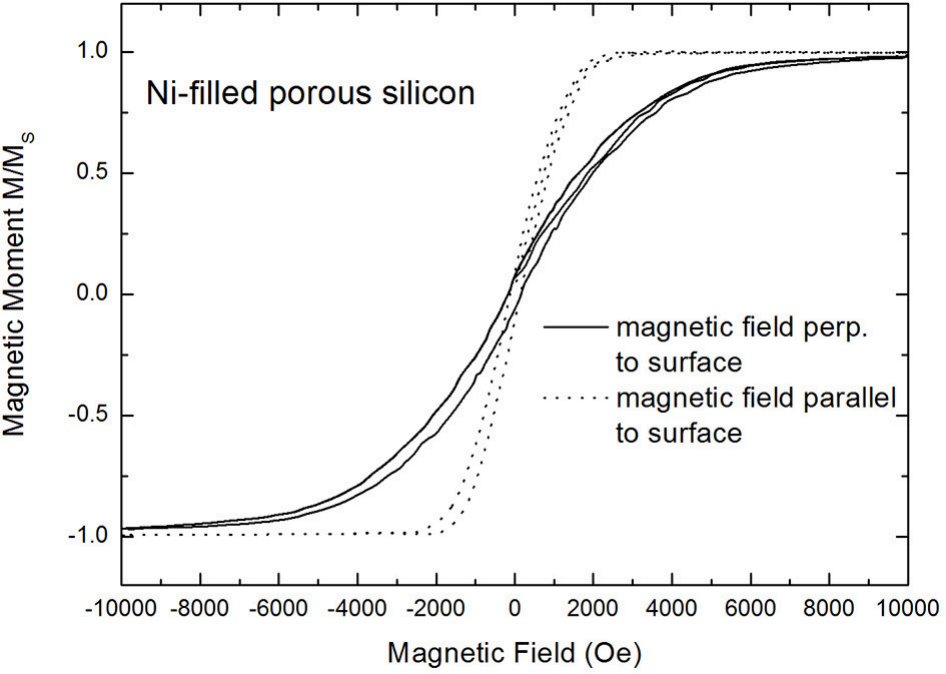
Magnetization curves performed with an external magnetic field applied parallel (dotted line) and perpendicular to the surface (full line), respectively. The measurements show a magnetic anisotropy between these two magnetization directions exhibiting the easy axis for an applied field parallel to the surface (Granitzer et al., 2018).

Field dependent magnetization measurements have been performed with a magnetic field applied parallel and perpendicular to the surface. In the case of isolated magnetic structures such measurements show a magnetic anisotropy which mainly depends on the shape anisotropy of the deposits. Elongated magnetic nanostructures offer a magnetic anisotropy with a magnetic easy axis along the long axis and a magnetic hard axis perpendicular to this one. The investigated samples offer in the case of the magnetic field applied perpendicular to the sample surface a low coercivity of about 200 Oe and a squareness (magnetic remanence/saturation magnetization) of about 0.2. Magnetization measurements performed with an external field applied parallel to the surface clearly show that the easy-axis of the sample coincides with this direction. Figure 6 shows the magnetic anisotropy between these two magnetization directions with the easy axis for an applied external magnetic field parallel to the surface. It can be seen that in the case of a parallel applied magnetic field the saturation magnetization is reached at about 1,000 Oe, whereas in the case of a perpendicular applied field to the sample surface the saturation magnetization is reached at about 9,000 Oe.

**Figure 7:**
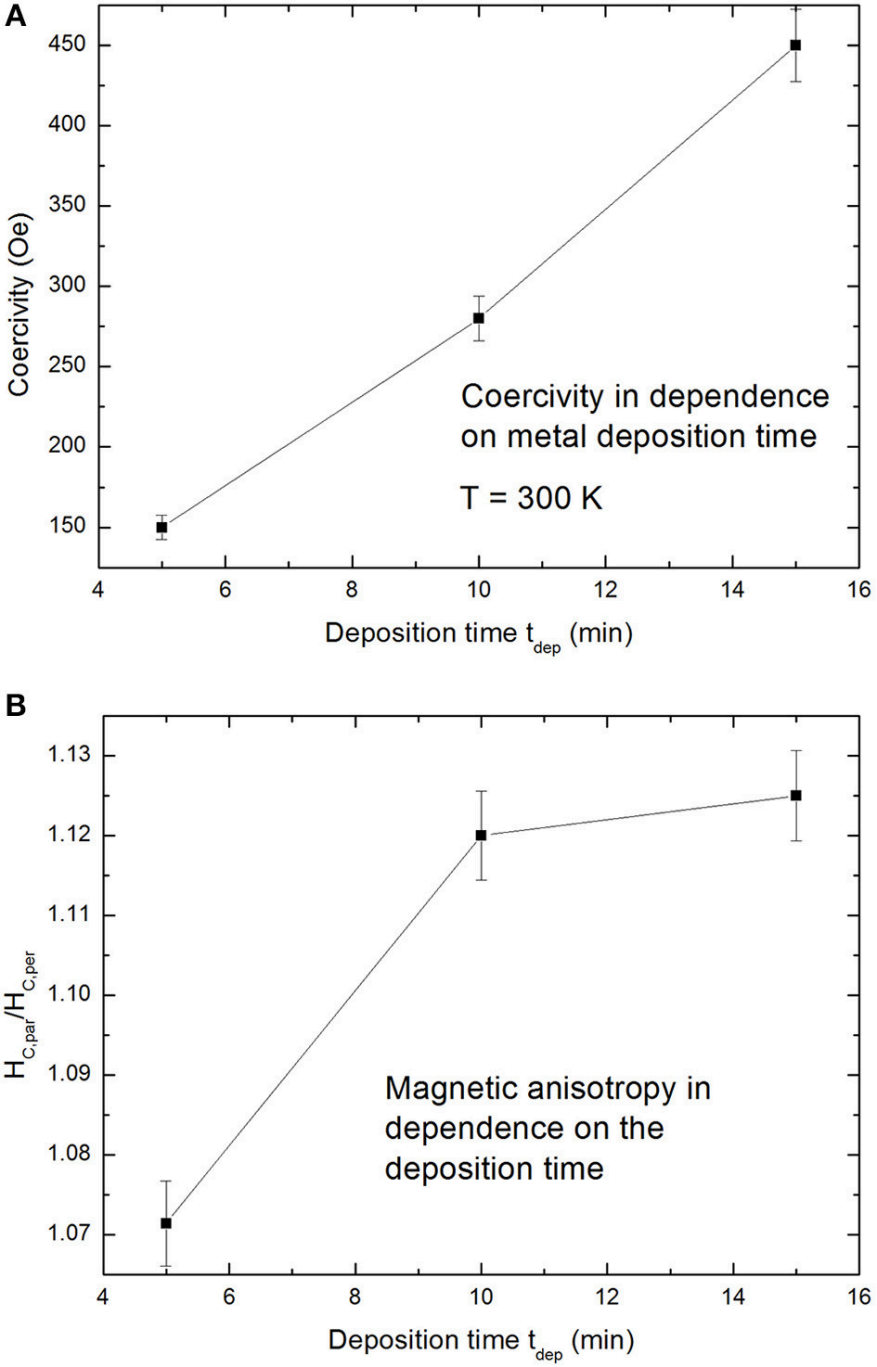
**(A)** The coercivity, obtained from measurements with an applied field parallel to the surface, in dependence on the deposition time. **(B)** The magnetic anisotropy (H_C,par_/H_C,per_) in dependence on the deposition time (Granitzer et al., 2018).

The film-like behavior, offering an in-plane easy axis within the film, shown in Figure 6 is caused by the interconnected Ni structures due to the branched morphology of the porous silicon template. Figure 7A shows an increase of the coercivity with increasing metal deposition time, whereat the measurements have been performed with an applied magnetic field parallel to the surface. The dependence of the magnetic anisotropy on the deposited Ni amount within the pores is presented in Figure 7B.

The optical properties, especially the photoluminescence and the decay times are varied by the amount of metal deposits within the porous silicon layer. The deposition of Ni within the porous silicon leads to a blue shift of the photoluminescence peak. Furthermore, an increase of the metal deposition time results in an enhancement of the photoluminescence intensity and a decrease of the photoluminescence life time which can be interpreted by a decrease in the radiative life time. The enhancement of the photoluminescence intensity is interpreted by the coupling of the silicon emitter to the Ni plasmons. Due to the direct vicinity of the emitter and the metal structure, only separated by a thin SiO_2_ native layer, coupling of the plasmonic modes with the emitter can occur. The magnetic properties of the Ni filled samples also show a dependence on the amount of metal deposits. An increase of the deposition time meaning an increase of the metal amount within the porous layer leads to more interconnected metal deposits. This behavior is evidenced by the enhancement of the coercivity from about 150 Oe to about 450 Oe with increasing deposition time from 5 to 15 min. Furthermore, a magnetic film-like behavior is observed which becomes more distinct with increased metal amount due to more interconnections. A magnetic anisotropy between the two magnetization directions parallel and perpendicular to the sample surface is observed which is typically for a magnetic film. In this case the magnetic easy axis corresponds to the parallel direction.

## Conclusions

In the frame of this work the optical characteristics of luminescent PSi compared with Ni filled porous silicon is discussed especially with respect to the position of the photoluminescence peak and its intensity. The PL peak is blue-shifted and the luminescence intensity is increased with increasing metal filling within the pores which is attributed to the coupling of the silicon emitter with the plasmons of the metal structures. Field dependent magnetization measurements of the nanocomposites are performed with an applied field parallel and perpendicular to the sample surface. A strong magnetic anisotropy between these two magnetization directions is observed, showing the easy axis for an applied field parallel to the surface. This film-like magnetic behavior is due to the interconnected metal structures which are present because of the branched morphology of the porous silicon. The coercivity of the samples decreases with decreasing metal filling and approximates to a superparamagnetic behavior which occurs in the case of separated magnetic nanoparticles. The presented systems which merge optical and magnetic properties are of interest for optoelectronics and magneto optical integrated devices.

## Author Contributions

KR and PG fabricated the porous silicon samples by anodization and subsequent electrodeposition and carried out the optical and magnetization measurements. PP performed the SEM investigations. MR supported the work with discussions. All authors discussed the data and prepared the manuscript.

### Conflict of Interest Statement

The authors declare that the research was conducted in the absence of any commercial or financial relationships that could be construed as a potential conflict of interest.
